# Familial frontotemporal dementia with neuronal intranuclear inclusions is not a polyglutamine expansion disease

**DOI:** 10.1186/1471-2377-6-32

**Published:** 2006-08-31

**Authors:** Ian R Mackenzie, Stefanie L Butland, Rebecca S Devon, Emily Dwosh, Howard Feldman, Caroline Lindholm, Scott J Neal, BF Francis Ouellette, Blair R Leavitt

**Affiliations:** 1 Department of Pathology, University of British Columbia, Vancouver, BC, Canada; 2 UBC Bioinformatics Centre, Department of Medical Genetics, University of British Columbia, Vancouver, BC, Canada; 3 Medical Genetics Section, University of Edinburgh, Molecular Medicine Centre, Western General Hospital, Edinburgh, UK; 4 Division of Neurology, Department of Medicine, University of British Columbia, Vancouver, BC, Canada; 5 Centre for Molecular Medicine and Therapeutics, Department of Medical Genetics, University of British Columbia, Vancouver, BC, Canada

## Abstract

**Background:**

Many cases of frontotemporal dementia (FTD) are familial, often with an autosomal dominant pattern of inheritance. Some are due to a mutation in the tau- encoding gene, on chromosome 17, and show an accumulation of abnormal tau in brain tissue (FTDP-17T). Most of the remaining familial cases do not exhibit tau pathology, but display neuropathology similar to patients with dementia and motor neuron disease, characterized by the presence of ubiquitin-immunoreactive (ub-ir), dystrophic neurites and neuronal cytoplasmic inclusions in the neocortex and hippocampus (FTLD-U). Recently, we described a subset of patients with familial FTD with autopsy-proven FTLD-U pathology and with the additional finding of ub-ir neuronal intranuclear inclusions (NII). NII are a characteristic feature of several other neurodegenerative conditions for which the genetic basis is abnormal expansion of a polyglutamine-encoding trinucleotide repeat region. The genetic basis of familial FTLD-U is currently not known, however the presence of NII suggests that a subset of cases may represent a polyglutamine expansion disease.

**Methods:**

We studied DNA and post mortem brain tissue from 5 affected members of 4 different families with NII and one affected individual with familial FTLD-U without NII. Patient DNA was screened for CAA/CAG trinucleotide expansion in a set of candidate genes identified using a genome-wide computational approach. Genes containing CAA/CAG trinucleotide repeats encoding at least five glutamines were examined (n = 63), including the nine genes currently known to be associated with human disease. CAA/CAG tract sizes were compared with published normal values (where available) and with those of healthy controls (n = 94). High-resolution agarose gel electrophoresis was used to measure allele size (number of CAA/CAG repeats). For any alleles estimated to be equal to or larger than the maximum measured in the control population, the CAA/CAG tract length was confirmed by capillary electrophoresis. In addition, immunohistochemistry using a monoclonal antibody that recognizes proteins containing expanded polyglutamines (1C2) was performed on sections of post mortem brain tissue from subjects with NII.

**Results:**

No significant polyglutamine-encoding repeat expansions were identified in the DNA from any of our FTLD-U patients. NII in the FTLD-U cases showed no 1C2 immunoreactivity.

**Conclusion:**

We find no evidence to suggest that autosomal dominant FTLD-U with NII is a polyglutamine expansion disease.

## Background

Frontotemporal dementia (FTD, OMIM: 600274) is a neurodegenerative disease characterized by abnormalities in personality, behaviour and language with relative early preservation of episodic memory [1,2]. The pathology underlying clinical FTD is heterogeneous [3]. In some cases, post mortem examination discloses abnormal accumulations of the microtubule associated protein tau in neurons and/or glial cells. However, several recent studies have demonstrated that the most common pathology associated with clinical FTD is the presence of dystrophic neurites and neuronal cytoplasmic inclusions in the cerebral cortex and hippocampus that are immunoreactive for ubiquitin (ub-ir) but negative for tau, synuclein and intermediate filament proteins (FTLD-U) [4,5].

FTD is often familial, usually with an autosomal dominant pattern of inheritance. Various studies have demonstrated genetic linkage to loci on chromosomes 3, 9 and 17 [6-11]. The gene on chromosome 3 and one of the genes on chromosome 9 have recently been identified (chmp2B and valosin-containing protein, respectively) [12,13]. Some cases that show linkage to chromosome 17 are found to have mutations in the gene for tau (*MAPT*) and all such cases show tau pathology [14,15]. Familial FTLD-U has been linked to several different loci on chromosome 9 and to chromosome 17q21 [6,7,10,11,16]. Interestingly, the 17q21 locus contains *MAPT*, and yet these cases do not have any tau pathology and have not had any tau mutations identified [6,7,11]. It remains unclear whether these cases are due to some, as yet, unrecognized abnormality in *MAPT *or whether there is another gene on 17q21 that is responsible for familial FTLD-U.

Recently, we reported that a subset of patients with familial FTLD-U have the additional post mortem finding of unusual, lentiform, ub-ir neuronal intranuclear inclusions (NII) [11,17]. At least some of the cases of familial FTLD-U with NII are ones that have shown linkage to the 17q21 locus [6,7,11]. NII are uncommon in neurodegenerative disease in general, but are a characteristic pathological feature of several conditions for which the genetic basis is abnormal expansion of a polyglutamine-encoding CAG trinucleotide repeat within the gene (examples include Huntington's disease (HD) and several types of spinocerebellar ataxia (SCA) [18-20]. The NII observed in these diseases are composed in part of aggregates of the expanded-repeat proteins. In this study, we explore the possibility that autosomal dominant FTLD-U with NII is a polyglutamine expansion disease.

## Methods

### Study cohort

**Figure 1:**
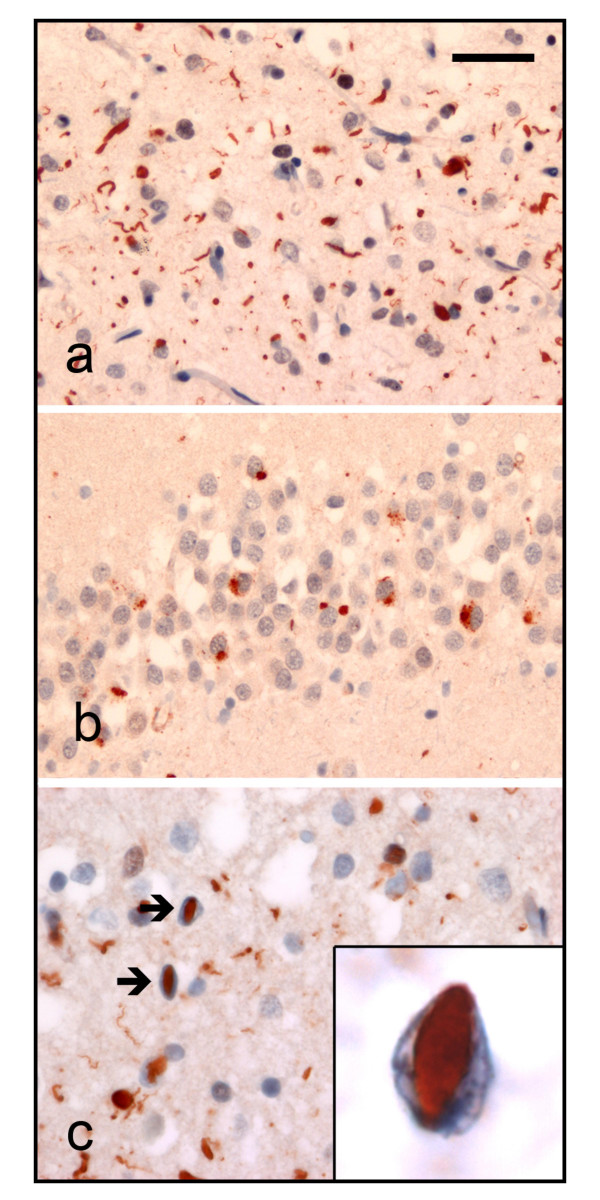
**Neuropathologic analysis of brain tissue from FTLD-Upatients**. Ubiquitin immunohistochemistry in cases of familial FTLD-U demonstrates staining of (a) neurites and neuronal cytoplasmic inclusions in the superficial cerebral neocortex, (b) neuronal cytoplasmic inclusions in hippocampal dentate granule cells, and (c) neuronal intranuclear inclusions in the cerebral neocortex (arrows). Scale bar; (a) and (b) 40 μm, (c) 25 μm, insert 6 μm.

Six patients with familial FTD, from five different families, were included in the study. In all families studied here, the pattern of inheritance suggested an autosomal dominant trait with a high degree of penetrance as previously described [11,17]. There was no evidence of genetic anticipation in any of the families. Five of the study patients were deceased and each was from a different family. Post-mortem neuropathological examination confirmed the presence of ub-ir neuronal cytoplasmic inclusions characteristic of FTLD-U [Figure 1 a,b] and an absence of significant tau and α-synuclein pathology (not shown). In four deceased patients (cases 1–4), numerous ub-ir NII were also observed. NII had a characteristic lentiform shape and were most numerous in small neurons of the frontotemporal neocortex and striatum [Figure 1c] [17]. The fifth deceased patient had FTLD-U type pathology but no NII (case 6). One living patient with FTD was also included in the study; he was the cousin of one of the deceased study patients with FTLD-U and NII (case 5). Therefore, the six study patients represented four families with FTLD-U with NII (cases 1–5) and one family with FTLD-U without NII (case 6).

### Immunohistochemistry

Post mortem brain tissue from the four deceased study patients with previously confirmed NII was evaluated. Formalin fixed, paraffin-embedded tissue sections from frontal cortex and striatum (areas with maximal numbers of NII), were immunostained using the Ventana ES automated system (Ventana, Tuscon, AZ) with primary antibodies against ubiquitin (DAKO, anti-ubiquitin; 1:500, following microwave antigen retrieval) and proteins with expanded polyglutamine domains (Chemicon, 1C2; 1:100 following formic acid pre-treatment). Tissue sections from two patients with known polyglutamine expansion diseases (one HD and one SCA1) were included as positive controls.

### Genetic analysis of polyglutamine-encoding genes

Using a computational approach, we have previously identified 63 genes in the human genome that contain tracts of CAG/CAA trinucleotide repeats which encode at least five consecutive glutamines (Butland *et al*., in submission). FTLD-U patient DNA was screened for expansions in these trinucleotide repeat tracts (n = 64), including the nine tracts whose expansion is currently known to cause neurodegenerative disorders in humans. One gene (PCQAP) contains two distinct polyglutamine-encoding tracts that were screened separately, hence the disparity in tract number versus gene number.

Gene-specific PCR primers were used to amplify the repeat-containing loci from the patient DNA, and the amplicons were subjected to high-resolution gel electrophoresis to measure allele size. Metaphor agarose gels (3% w/v, Mandel Scientific) were run overnight at low voltage in re-circulated buffer. Under such conditions, it is possible to resolve small allelic differences [see Additional file 1]. Gel data were digitised and interpreted with IMAGE^® ^software, and product sizing was estimated to be accurate within 6 base pairs. The length of CAG/CAA tracts was inferred from the overall allele length based on calibration data we obtained by directly sequencing several alleles from each locus. In no case did we observe allele length differences that arose from sequence changes outside of the repeat tract. Thus, all allele size changes correspond to differences in the repeat tract alone. From the present data we cannot, however, determine where in the repeat tract, such as in the longest contiguous CAG tract, the expansion is occurring.

The data presented herein refer to the longest uninterrupted tract of CAG and CAA trinucleotides in the given gene. These values were compared to published data (for known disease loci) and to data obtained from a control group (n = 94) of unaffected individuals (Butland *et al*., in submission). Based on the above resolution limit, all alleles estimated to be 6 or more b.p. longer than the longest control allele were subjected to confirmatory capillary electrophoresis on an ABI 3700 DNA Analyzer (Applied Biosystems) and subsequent analysis using GeneMapper software (Applied Biosystems).

This research was carried out in compliance with the Helsinki Declaration, and ethical approval was provided by the Clinical Research Ethics Board of the University of British Columbia (certificate C03-0449).

## Results

### Neuropathologic analysis of FTLD-U brain tissue

Ubiquitin-positive neurites and neuronal cytoplasmic inclusions were identified in the superficial cerebral neocortex of the FTLD-U patients (Figure 1a), and similar neuronal cytoplasmic inclusions were found in hippocampal dentate granule cells (Figure 1b). Ub-ir NII were found in the cerebral neocortex (arrows, Figure 1c). NII in the control cases with known polyglutamine disease (HD and SCA1) were immunoreactive for both ubiquitin and 1C2. In contrast, NII in FTLD-U patients were ub-ir, but were 1C2 negative (data not shown).

### Genetic analysis of FTLD-U DNA samples for CAG/CAA repeat expansions in polyglutamine-encoding genes

FTLD-U patient DNA was screened for expansions in the CAA/CAG trinucleotide repeat tracts of our candidate genes (Table 1), and the repeat sizes from the study subjects were compared with published normal values and with those of healthy controls (n = 94) (Butland *et al*., in submission). Sixteen small putative expanded alleles were detected, while the vast majority of alleles from our FTD patients fell within the range of normal alleles from our unaffected controls. At least one allele was identified in each patient that fell just outside the range observed in our controls samples, and these alleles arose from eight genes (CACNA1A, ARID3B, BAIAP1, C9orf43, EP400, FOXP2, MAML2, PAXIP1L). The largest putative expansion identified consisted of a 15 b.p. (~ 5 CAG/CAA) increase over the largest control allele (case 2, MAML2). The exact CAG repeat size of all the putative expanded alleles was confirmed by capillary electrophoresis sizing and/or direct DNA sequencing, and none of these specific alleles was found to be expanded in all of the affected FTDL-U individuals.

## Discussion

We have adapted an inexpensive, high-resolution agarose gel electrophoresis method for the precise sizing of targeted polyglutamine-encoding repeat tracts in the human genome. Using this method, the normal distribution of CAG repeat lengths for known disease loci compared favourably with previously published data and matched the results obtained by direct sequencing of specific alleles, thus confirming the reliability of our method. No significant CAA/CAG repeat expansions were detected at the nine known disease loci in any of the FTLD-U patients. The initial analysis of the data suggested a putative minor expansion in the CACNA1A gene (case 1) by gel electrophoresis that was subsequently refuted by capillary electrophoresis. This method was subsequently applied to 55 candidate loci that we identified as part of our computational screen for polyglutamine-encoding tracts in the human genome (Butland *et al*., in submission). We identified 15 alleles from seven genes in our FTLD-U patients that were 6 or more b.p. longer than the longest control allele (Table 1, allele sizes in bold text). None of these small expansions consistently correlated with affected FTLD-U status in multiple individuals. An allele of MAML2 harboured the largest putative expansion (15 b.p., case 2). However, according to the annotation of the reference genome, the trinucleotide repeat tract passes through a proposed intron. Thus, it is unclear if in fact this allele would encode an expanded polyglutamine tract in the protein.

Several factors make it unlikely that the small putative expansions identified are pathogenic. First, there were no genes for which the allele sizes in FTD patients with NII (cases 1–5) were consistently larger than those from the FTD patient without NII (case 6). Second, pathogenic alleles of known human polyglutamine disorders typically encode more than 35 consecutive glutamines whereas none of our putative expanded alleles encode this many.

Our histopathological findings also support the conclusion than FTLD-U with NII is not a polyglutamine expansion disorder. All known polyglutamine expansions disorder include the presence of ub-ir NII which also label with the monoclonal antibody 1C2 [18-20]. The absence of any 1C2 reactivity in our FTLD-U patient tissue thus makes it unlikely that their NII contain proteins with expanded polyglutamine tracts.

Finally, although only a small number of such families have been reported to date, there is evidence that NII may be a specific pathological marker for FTLD-U linked to the chromosome 17q21 locus [6,7,11]. Our computational approach did not identify any gene in the defined region of interest that contains at least five CAG or CAA repeats, although ubiquitinated intranuclear inclusions are known to occur in triplet repeat disorders encoding for amino acids other than glutamine (alanine), and in disorders caused by expansions in untranslated regions. This region does contain 294 non-CAG trinucleotide repeat tracts of minimum length 5; 11 of which are found within the coding sequences of 6 known and 3 hypothetical genes [21]. Thus, a non-glutamine encoding trinucleotide repeat expansion could still be the basis of the observed phenotype.

## Conclusion

In summary, we find no evidence to suggest that autosomal dominant FTLD-U with NII is a polyglutamine expansion disease. We did not observe immunoreactivity for expanded polyglutamines within FTLD-U brain, nor did we identify any alleles with large polyglutamine-encoding repeat expansions in our set of candidate genes, which comprises all of the predicted genes of interest with at least five polyglutamine-encoding CAA/CAG repeats in the human genome. Furthermore, none of the slightly longer alleles from FTLD-U subjects within the candidate genes identified by our computational approach are found within the various linkage regions established in prior studies. Therefore, both our genetic analysis and immunohistochemical data, suggest that the formation of NII in FTLD-U is due to a mechanism other than accumulation of a protein with a polyglutamine expansion.

## Competing interests

The author(s) have no competing interests to disclose.

## Authors' contributions

SLB, RSD, BRL, BFFO, IRM conceived and designed the experiments. HF, CL, ED contributed the genetic and clinical materials. SLB, RSD, IRM performed the experiments. SLB, SJN, IRM analysed the data. SLB, SJN, IRM and BRL wrote the paper and all other authors provided comments.

## Note added in proof

The conclusions of this manuscript have recently been verified by two manuscripts: Baker M *et al*, Mutations in progranulin cause tau-negative frontotemporal dementia linked to chromosome 17. Nature. 2006 Aug 24;442(7105):916–9, and Cruts M, *et al*. Null mutations in progranulin cause ubiquitin-positive frontotemporal dementia linked to chromosome 17q21. Nature. 2006 Aug 24;442(7105):920–4. This work identified non-polyglutamine encoding mutations in the gene encoding progranulin as the cause of FTLD-U in the subjects we studied.

## Pre-publication history

The pre-publication history for this paper can be accessed here:



## Supplementary Material

Additional File 1 **A comparison of the agarose gel method of CAG repeat allele size measurement with conventional capillary electrophoresis**. We have proven the reliability of the agarose gel method by comparing product sizes generated using this technology with results from an ABI PRISM^® ^3100 Genetic Analyzer, and also by direct DNA sequencing of the PCR products. As positive controls for larger fragments, we have also performed this comparison on a set of DNA samples known to harbour an expansion in the HD gene (Supplemental Figure 1). Electrophoresis of a PCR product with a mono-allelic expansion in the HD repeat, by agarose gel electrophoresis (panel A) and by ABI Genetic Analyzer (panel B). The location of the two alleles is marked by green dots (panel A) or blue peaks (panel B); the dark band with no green dot is the well of the agarose gel. The difference in CAG repeat length between the 'normal' and the 'expanded' allele is identical between both methods. Click here for file

## Figures and Tables

**Table 1:** Genetic analysis of FTLD-U DNA samples reveals no significant CAG/CAA repeat expansions in candidate polyglutamine-encoding genes.

	**Gene Name (associated polyQ disease, CAA/CAG tract length of smallest disease-cuasing allele) ^a^**	**Control Samples (n = 94) estimated CAA/CAG tract length**	**FTD Patients estimated CAA/CAG tract length**					
			
			**case 1^b^**	**case 2^b^**	**case 3^b^**	**case 4^b^**	**case 5^b^**	**case 6^c^**
**A**	**AR (SBMA, 38)**	10–37	22	19	20	21, 23	19	24
	**ATN1 (DRPLA, 48)**	10–39	18, 23	18, 20	20, 23	19	15, 19	15
	**ATXN1 (SCA1, 39)**	6–39	15, 20	15, 20	16	15, 20	15, 20	14, 21
	**ATXN2 (SCA2, 37)**	25–41	22	22	22	22	22	22
	**ATXN3 (SCA3/MJD, 51)**	14–42	15	9	9	9	17	na
	**ATXN7 (SCA7, 36)**	4–35	10	11	10	10	10	10
	**CACNA1A (SCA6, 20)**	4–18	12, **21^d^**	13, 16	15	14	14	13
	**HD (HD, 36)**	10–35	14, 15	12, 17	17, 21	15, 17	12, 14	12
	**TBP (SCA17, 49)**	38–61	39, 44	38, 41	38, 43	36, 39	38, 47	38, 42
								
**B**	ARID3B	11–13	12	12	12, **16**	12, **16**	13, **16**	13
	ASCL1	5–24	13, 17	13	13, 17	13, 18	13, 18	12
	BMP2K	22–31	26	26	26	26	26	26
	C14orf4	19–24	22, 25	22, 25	23	23	24	23
	CXorf6	11–12	12	12	12	11	11	12
	DCP1B	10–12	12	na	12	11, 12	11, 12	11, 12
	KCNN3	10–43	18	17, 18	na	19, 21	18, 21	18, 20
	MED12	26	27	26	26	26	26	26
	MEF2A	9–16	12, 15	11	12	10, 11	11, 14	11
	MINK1	5–6	6	6	6	6	6	7
	MLL2	6–9	9	9	9	9	9	9
	NCOA3	25–34	26, 29	28	26, 28	29	28, 29	28
	NCOA6	23–26	25	25	na	24	25	25
	NCOR2	13–22	17, 19	17	17	16, 17	13, 17	17, 18
	NFAT5	18–21	19	18	18	18	na	na
	NM 014856	13–17	16	17	14, 16	17	16	14, 16
	NUMBL	26	18, 20	18	20	18, 20	18, 20	20
	PCQAPa	10–18	11	11	11	10	10	11
	PCQAPb	11–22	17	16	16	15	16	16
	PHLDA1	15–19	15	15	16	15	15	15
	POLG	13–16	13	14	13	16, 17	13	13
	POU3F2	20–21	15	21, 22	21	21	21	21
	POU6F2	7–11	10	10	10	10	10	10
	PRDM10	7–8	8	8	8	8	8	8
	PRKCBP1	7–11	8	8	7	8	8	8
	RAI1	8–20	14	14	14	14	14	10, 14
	RUNX2	26–53	22	21	22	23	23	21
	SATB1	12–21	15	15	15	15	15	15
	SMARCA2	18–25	22	22	22	22	22	22
	SOCS7	7–22	8, 12	8	8	8	8	8
	TFEB	9–15	12	9	9	9	10	10
	TNRC4	14–17	15	15	15	15	15	15
	TNRC6B	7–10	9, 10	9	9	9	8	8
	TNS	8–11	10, 12	10	9	10	9	10
	ZNF161	8–22	16, 17	14, 20	14	na	14	14
	ZNF384	11–20	15	16	15	15	20	15
								
**C**	ARID1B	16–23	18	18	19	19	18	18
	BAIAP1	16–21	20, **24**	19, **25**	18, 19	19	20	17, 19
	BRD4	8–9	8	8	8	8	8	8
	C9orf43	8–9	9	9, **12**	8	8	9, **12**	8
	CHERP	12	12	12	12	12	12	12
	CIZ1	6	6	6	6	6	6	6
	CREBBP	18	18	18	18	18	18	18
	EP400	28–31	30	30, **34**	30	30	31	30, **34**
	FOXP2	34–40	41	na	41	41	42	42
	KIAA1817	26–27	27	28	28	28	27	27
	KIAA2018	11–16	13	14	14	14	12, 17	12
	MAML2	27–31	29	32, **36**	28	28	28, 31	28
	MAML3b	18	19	19	19	19	19	19
	MN1	26–30	28	28	28	28	28	28, 29
	PAXIP1L	7	**9**	**9**	**9**	8	8	8
	PHC1	13–15	15	15	15	15	15	15
	ST6GALNAC5	12–14	12	12	12	12	12	12
	THAP11	18–30	24	24	24, 33	24	24, 26	24, 28
	TNRC6A	4–8	4, 8	5, 8	8	8	5, 8	8

^a ^Genes identified in a computational analysis of polyglutamine repeat-containing genes in the human genome. A) Genes known to cause disease via polyglutamine expansion. B) Patient and control samples were both assessed by high resolution agarose gel electrophoresis. C) Control samples were analyzed by capillary electrophoresis (Butland *et al*., in submission) and patient samples analyzed by high resolution agarose gel electrophoresis.

^b ^FTDL-U patients with NII.

^c ^FTDL-U patient without NII.

^d ^Alleles 6 or more b.p. longer than the largest contol allele (putative expanded alleles) appear in bold type.

Samples of FTLD-U patient DNA with NII (Cases 1–5) and without NII (Case 6) were screened for expansions in the CAG/CAA trinucleotide repeat tracts of our candidate genes including known disease genes (**A**). The CAG/CAA repeat lengths from the FTLD-U subjects were compared with published normal values and with those of healthy controls (n = 94), assessed by high resolution agarose gel electrophoresis (**B**), and/or capillary electrophoresis (**C**). For the few alleles estimated to be equal to or slightly larger than the maximum measured in our control samples (**bold**), the CAG/CAA repeat length was confirmed by capillary electrophoresis on an ABI 3700 sequencer using GeneMapper software. Using this approach, no clinically significant CAG/CAA repeat expansions were identified in the DNA from any of our FTLD-U patients.
