# Supplementary material for: Familial frontotemporal dementia with neuronal intranuclear inclusions is not a polyglutamine expansion disease
Source: BMC Neurol. 2006 Aug 31;6:32. doi: 10.1186/1471-2377-6-32 (PMC1570137; doi:10.1186/1471-2377-6-32)
Supplement: Additional File 1 — A comparison of the agarose gel method of CAG repeat allele size measurement with conventional capillary electrophoresis. We have proven the reliability of the agarose gel method by comparing product sizes generated using this technology with results from an ABI PRISM® 3100 Genetic Analyzer, and also by direct DNA sequencing of the PCR products. As positive controls for larger fragments, we have also performed this comparison on a set of DNA samples known to harbour an expansion in the HD gene (Supplemental Figure 1). Electrophoresis of a PCR product with a mono-allelic expansion in the HD repeat, by agarose gel electrophoresis (panel A) and by ABI Genetic Analyzer (panel B). The location of the two alleles is marked by green dots (panel A) or blue peaks (panel B); the dark band with no green dot is the well of the agarose gel. The difference in CAG repeat length between the 'normal' and the 'expanded' allele is identical between both methods. [file 1471-2377-6-32-S1.ppt]

## Slide 1
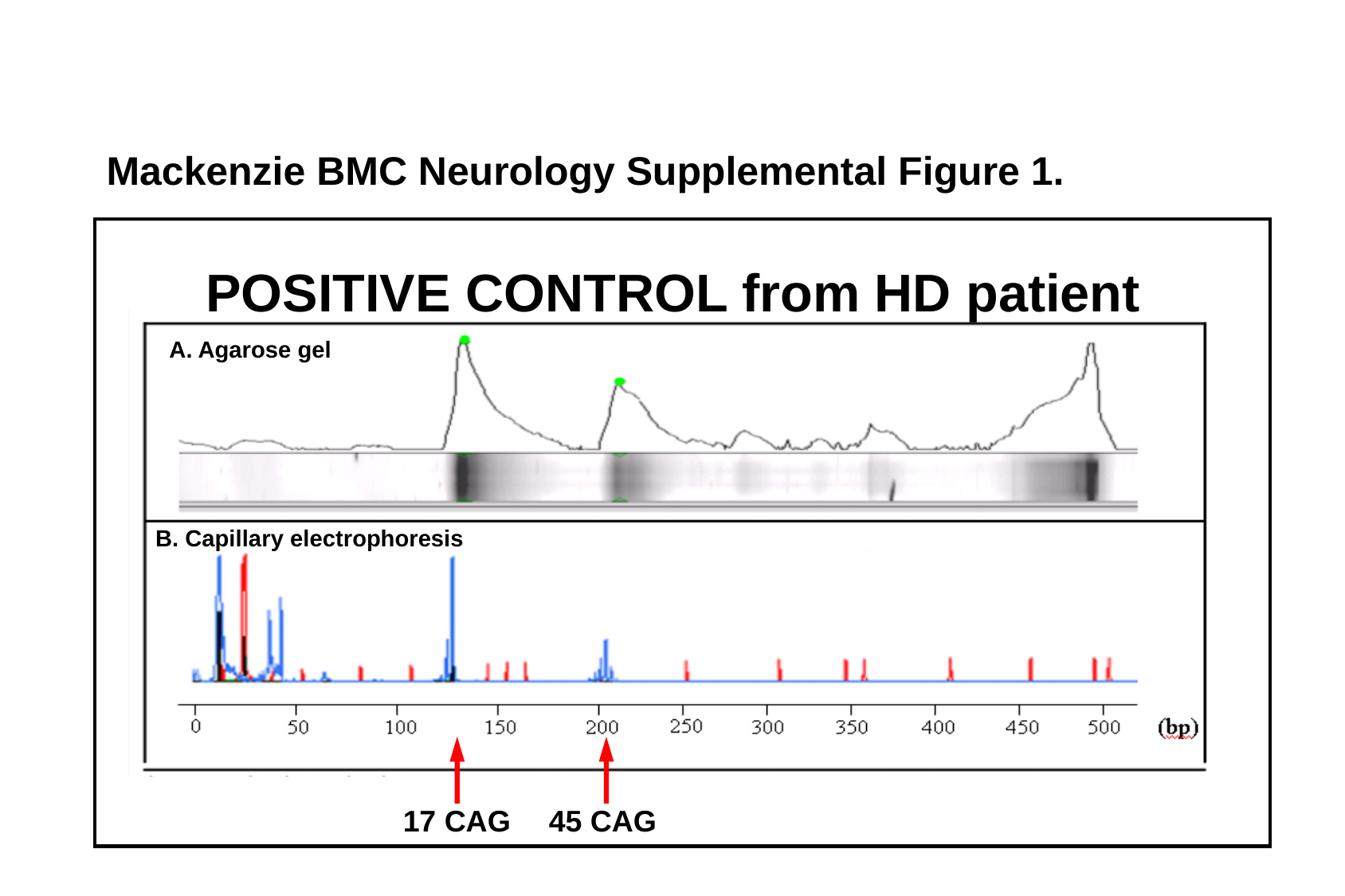

Mackenzie BMC Neurology Supplemental Figure 1.
POSITIVE CONTROL from HD patient
A. Agarose gel
B. Capillary electrophoresis
17 CAG
45 CAG
